# Association Between Impairment of DNA Double Strand Break Repair and Decreased Ovarian Reserve in Patients With Endometriosis

**DOI:** 10.3389/fendo.2018.00772

**Published:** 2018-12-21

**Authors:** Young Sik Choi, Ji Hyun Park, Jae Hoon Lee, Jeong-Kee Yoon, Bo Hyon Yun, Joo Hyun Park, Seok Kyo Seo, Hak-Joon Sung, Hyun-Soo Kim, SiHyun Cho, Byung Seok Lee

**Affiliations:** ^1^Department of Obstetrics and Gynecology, Severance Hospital, Yonsei University College of Medicine, Seoul, South Korea; ^2^Institute of Women's Life Medical Science, Yonsei University College of Medicine, Seoul, South Korea; ^3^Department of Obstetrics and Gynecology, Gangnam Severance Hospital, Yonsei University College of Medicine, Seoul, South Korea; ^4^Department of Medical Engineering, Yonsei University College of Medicine, Seoul, South Korea; ^5^Department of Pathology, Severance Hospital, Yonsei University College of Medicine, Seoul, South Korea

**Keywords:** endometriosis, ovarian reserve, double stranded DNA break, BRCA1 gene, gamma-H2AX

## Abstract

**Background:** Repair of DNA double strand break (DSB) is an important mechanism for maintaining genetic stability during a DNA damage event. Although, a growing body of recent evidence suggests that DNA DSBs and related repair mechanisms may be important in ovarian aging and in various cancers, there are few reports in endometriosis. We, therefore, examined expression levels of genes pertaining to DNA DSB repair in patients with endometriosis to assess the potential effects on ovarian reserves.

**Materials and methods:** A total of 69 women undergoing laparoscopic surgery for endometriosis and other benign conditions was included; endometriosis group (*n* = 38) vs. controls (*n* = 31). DNA DSBs in endometrial and ovarian tissues of both groups were compared via immunohistochemistry, aimed at γ-H2AX expression. To gauge genotoxin-induced DNA DSBs in endometrial stromal cells, γ-H2AX expression was determined by western blot after H_2_O_2_ treatment of cultured endometrial stromal cells (endometriosis group and controls) and Ishikawa cell-line cultures. Endometrial and ovarian tissue levels of BRCA1, BRCA2, Rad51, and ATM (ataxia-telangiectasia mutated) mRNA expression were also compared. Correlations between expression levels of genes of interest and serum anti-müllerian hormone (AMH) levels were assessed as well.

**Results:** Expression of γ-H2AX in immunostained endometrial and ovarian tissue preparations was greater in the endometriosis group, compared with controls. After H_2_O_2_ treatment, γ-H2AX expression levels were also significantly greater in cultured stromal cells of the endometriosis group and in the Ishikawa cell line than in controls. Endometrial expression of *BRCA1* and *Rad51* mRNA proved significantly lower in the endometriosis group (vs. controls), as did ovarian expression of *BRCA1* and *BRCA2* mRNA. Serum AMH concentration showed a significant correlation with ovarian *BRCA1* mRNA expression in women with endometriosis (*p* = 0.03).

**Conclusions:** In women with endometriosis, expression levels of various genes implicated in DSB repair are decreased and ovarian *BRCA1* expression correlates with

## Introduction

Endometriosis is characterized by the presence of endometrium-like epithelium and stroma outside the endometrium and myometrium (1), and not necessarily restricted to the pelvic compartment. This disease is one of the most common gynecologic disorders affecting ~10% of all reproductive-age women and 20–50% of women with chronic pelvic pain and/or infertility (2).

The biologic mechanisms that may link endometriosis and subfertility have not been fully explained. However, several mechanisms (e.g., pelvic adhesions, altered peritoneal, hormonal, or cell-mediated function, abnormal cytokine release, endocrine and ovulatory abnormalities, and impaired implantation) have been proposed (3). Studies from oocyte donation–*in vitro* fertilization and embryo transfer, which may exclude the impact of endometrial receptivity, suggest that subfertility in endometriosis is attributed to quality of oocytes rather than endometrial receptivity (4). In addition to the qualitative issues of oocytes, quantitative declines in ovarian reserve are also of concern for women with endometriosis (5). The impact of minimal-to-mild endometriosis on ovarian reserves has seemed inconsistent, but it is speculated that advanced-stage endometriosis may be detrimental in this regard, regardless of any surgical damage. As serum anti-Müllerian hormone (AMH) concentrations and antral follicle counts (AFCs) confirm, endometriomas *per se* are associated with diminished ovarian reserves (6, 7); and endometriosis or endometriomas may impact responsiveness to controlled ovarian stimulation, reflected by numbers of oocytes collected (8). Thus, declining ovarian reserves appear directly related to endometriosis. Such deterioration due to both pathologic underpinnings of endometriosis and any surgical damage incurred is the source of mounting attention on fertility preservation in women with endometriosis (5, 9).

At present, however, the pathophysiologic mechanisms of diminished ovarian reserves in endometriosis remain unclear. Tissues bordering endometriomas often show morphologic alterations (i.e., substantial loss of cortical stroma, fibrosis, and considerably less follicular density) that are not present near other benign cysts (10, 11). Similar pathologic changes, including focal fibrosis and vascular deficiency, have likewise been observed in ovarian cortex exposed to chemotherapy (12). The proportion of primordial follicles present in ovaries with endometriomas is distinctly lower by comparison, and there are notable increases in proportions of non-resting growing follicles (13). This “burn-out” effect on follicular reservoirs is also suggested as a major mechanism leading to chemotherapy-induced loss of ovarian reserves (14, 15). Hence, patients with endometriosis may follow a path similar to chemotherapy-induced ovarian damage.

Cells sustain DNA damage through both external and internal means. Among the various types of DNA damage due to environmental genotoxins, DNA double-strand breaks (DSBs) are capable of substantially altering genetic integrity and thus are the most deleterious. The ataxia-telangiectasia mutated (ATM)–mediated DNA damage signaling (DDS) pathway regulates repair of DNA DSBs via a homologous recombination mechanism. In instances of irreparable DNA damage, cells are eliminated by apoptotic cell death or undergo senescence (complete cell-cycle withdrawal) to avoid severe mutagenic consequences (16). Impaired DNA DSB repair may be associated with loss of ovarian follicular reserves, *BRCA1*, and other key genes in the ATM pathway that decline with age in human oocytes (17). Furthermore, chemotherapy causes massive DNA DSBs in primordial follicles, oocytes, and granulosa cells. Such damage is associated with apoptotic oocyte death, which then triggers the DNA repair response by activating the ATM-medicated DDS pathway (18, 19).

It has been suggested that endometriosis *per se* affects ovarian reserve. Although evidence of an association between DNA DSBs and ovarian reserves has emerged, there has been no pertinent data on women with endometriosis. Considering that it was reported that morphologic changes in ovary of women with endometriosis were similar to those after chemotherapy-induced ovarian damage, we hypothesized that diminished ovarian reserve in women with endometriosis may have similar pathway to chemotherapy-induced ovarian damage (e.g., increased DNA damage and impaired repair mechanism). In the present study, we assessed the extent of genotoxin-induced DNA damage to *in vitro* cultures of endometrial stromal cells. We also used endometrial and ovarian tissues of women with endometriosis to investigate DNA damage and expression levels of genes implicated in DNA DSB repair in an effort to determine the ramifications for ovarian reserves.

## Materials and Methods

### Study Population and Sample Collection

Among candidates undergoing laparoscopic surgery for various indications (e.g., pelvic masses or pain, endometriosis, infertility, and diagnostic evaluations of benign gynecologic diseases) from January 2015 to September 2017, only those aged 25–40 years with a body mass index (BMI) of 18.5–29.9 kg/m^2^ were included in this prospective case-control study after granting written informed consent. The present study was performed in university research center and approved by the Institutional Review Board of Gangnam Severance Hospital (IRB number 3-2015-0250). Postmenopausal status, previous use of a hormone or a gonadotropin-releasing hormone (GnRH) agonist within 6 months, prior ovarian surgery, or other medical disorders (including adenomyosis; endometrial hyperplasia, polyps, or cancer; infectious diseases; chronic or acute inflammatory diseases; malignancies; autoimmune diseases; or cardiovascular diseases) were grounds for study exclusion.

At the time of surgery, all possible endometriotic lesions were excised and sent for pathologic examination to confirm the diagnosis. Patients were assigned to the endometriosis group only after pathologic confirmation of the excised tissue. The extent of endometriosis was determined using the American Society of Reproductive Medicine (ASRM) revised classification (20). Overall, 38 patients displayed moderate-to-severe endometriosis (stages III and IV), histologically confirmed. Another 31 patients were confirmed that they had no endometriosis by laparoscopy and served as controls. They were diagnosed with the following ovarian neoplasms: dermoid cyst (*n* = 20), serous cystadenoma (*n* = 6), and mucinous cystadenoma (*n* = 5). Endometrial samples were collected from the patients by Pipelle sampler (Cooper Surgical, Trumbull, CT, USA) during surgery irrespective of menstrual phase. Tweleve out of 38 patients (31.5%) in the endometriosis group and 9 out of 31 patients (29.0%) in the controls were in proliferative phase and others in secretory phase.

Main outcomes were γ-H2AX protein expression, mRNA expression of BRCA1, BRCA2, and Rad51, and ATM in eutopic endometrium and ovarian tissue, γ-H2AX protein expression of cultured endometrial stomal cells after H2O2 treatment, and correlation between DNA DSB repair genes of interest and AMH.

### Immunohistochemistry

Immunohistochemical staining was performed in five participants of each group. γ-H2AX protein expression was assessed by ready-to-use immunostain application (Bond Polymer Intense Detection System; Vision BioSystems, Wetzlar, Germany) following the manufacturer's instructions. Surgically resected tissues were first fixed in 10% neutral buffered formalin for 12–24 h. Samples were then selected for routine processing (in automated system), embedding in paraffin, and slide preparation, sectioning at 4 μm by rotary microtome. Once deparaffinized (Bond Dewax Solution; Vision BioSystems), antigen retrieval proceeded (Bond Epitope Retrieval Solution; Vision BioSystems) by treating sections for 30 min at 100°C. Endogenous peroxidases were quenched by a 5-min hydrogen peroxide pretreatment. Sections were then incubated for 15 min at ambient temperature with rabbit polyclonal γ-H2AX antibody (1:500 dilution; Bethyl Laboratories, Montgomery, TX, USA). A biotin-free polymeric horseradish peroxidase-linked antibody conjugate system was finally applied (BOND-MAX automatic slide stainer; Vision BioSystems), and sections were developed using 1 mM 3,3′-diaminobenzidine as the chromogen, 50 mM Tris-hydrogen chloride buffer (pH 7.6), and 0.006% hydrogen peroxide, with hematoxylin as counterstain. Positive (breast cancer tissue), and negative control slides were generated for each reaction to minimize inter-assay variation. For the negative controls, the primary antibody was replaced by non-immune serum to yield no detectable γ-H2AX staining.

### Culture of Primary Endometrial Stromal Cells and Ishikawa Cell Lines

Since Ishikawa cell line, a well-differentiated human endometrial adenocarcinoma cell line bears estrogen and progesterone receptors, the cells have been used numerous basic research areas such as reproductive biology and molecular science including endometriosis researches (21). Since it was also suggested that genotoxic exposure such as chemotherapeutic agent induced substantial increase in gamma-H2AX in Ishikawa cell lines (22), these cell lines were used as a positive control to quantify DNA damage in *in vitro* cell culture study. Eutopic endometrium of women with endometriosis shows fundamental differences compared with that of healthy control (23). Since, therefore, eutopic endometrial stromal cells of women with endometriosis may have altered responses to genotoxic exposure, *in vitro* cell culture study was performed to compare the extent of DNA damage following genotoxic stimuli between endometrial stromal cells obtained from endometriosis group and controls.

We utilized a previously published method to culture endometrial stromal cells (24). Endometrium was finely minced, and the cells were dispersed by incubation at 37°C for 60 min with agitation while adding (pipetting) Hanks balanced salt solution (HBSS) containing 4-(2-hydroxyethyl)-1-piperazineethanesulfonic acid (HEPES; 2 mmol/mL), penicillin/streptomycin (1%), and collagenase (1 mg/mL, 15 U/mg). The cells were pelleted, washed, suspended in Ham's F12:Dulbecco's Modified Eagle Medium (DMEM) in a 1:1 ratio containing 10% fetal bovine serum (FBS) and 1% penicillin/streptomycin, passed through a 40-μm cell strainer (Falcon, Corning, NY, USA), and plated onto 75-cm^2^ Falcon tissue culture flasks (BD Biosciences, Bedford, MA, USA). Cultured primary human endometrial stromal cells (HESCs) at passages 3–5 were used for analysis. Ishikawa cells were maintained in MEM (Invitrogen, Carlsbad, CA, USA) containing 2.0 mmol/L l-glutamine and Earl's Salts, supplemented with 10% FBS, 1% sodium pyruvate, and 1% penicillin/streptomycin. HESCs from patients with endometriosis and Ishikawa cells were harvested from culture flasks using trypsin/EDTA (0.05%). The cells were then counted (5 × 10^6^) for plating in six-well plates at 37°C in a 5% CO_2_ humidified environment and grown as previously described. At 80% confluency, the cells were treated with 250 μmol/L H_2_O_2_ concentrations, extracting proteins 4 h later. *In vitro* cell culture experiments were triplicated.

### RNA Extraction and Quantitative Real-Time Polymerase Chain Reaction (RT-PCR)

Endometrial samples of all participants were analyzed by quantitative RT-PCR. Total RNA was extracted using a kit (RNeasy Mini; Qiagen, Valencia, CA, USA). A total of 2 μg RNA from each sample was reverse transcribed into cDNA (SuperScript III First-Strand Synthesis System; Invitrogen), all according to manufacturer protocols. Expression of candidate gene mRNA was measured by SYBR RT PCR on an ABI 7300 instrument (Applied Biosystems, Forster, CA, USA). We designed specific primers for BRCA1 (forward primer 5′-AGCTGTGTGGTGCTTCTGTGGT-3′, reverse primer 5′-TGGCTGCACAACCACAATTGGG-3′), BRCA2 (forward primer 5′-CTTGCTTTCAAATTGGCACTGA-3′, reverse primer 5′-GTTTAAAAGGGCATAGGCT CTG-3′), Rad51 (forward primer 5′-TAGC AAAGGGAATGGGT CTGC-3′, reverse primer 5′-GCACAAGACTCCATAACCAAAC-3′), and ATM (forward primer 5′-GCTCAGTGTTGGTGGACAGGT-3′, reverse primer 5′-TCCATCCTGGGAAAAGTCG GCT-3′). The PCR reaction was performed in 20 μL buffer containing 2 μL of cDNA, 5 pM each primer, and power SYBR green PCR master mix (Applied Biosystems). The thermal cycling conditions were pre-incubated for 2 min at 50°C, then denatured for 10 min at 95°C, followed by 40 cycles of denaturation for 15 s at 95°C, and annealing and extension for 1 min at 60°C. To normalize the amount of total RNA present in each reaction, we amplified the housekeeping gene, glyceraldehyde-3-phosphate dehydrogenase (GADPH). Sequences for GAPDH were as follows: forward primer 5′-GAAGGT GAAGGTCGGAGTC-3′ and reverse primer 5′-GAAGATGGTGATGGGATTTC-3′. The amount of target, which was normalized to the endogenous reference (GAPDH) and was compared to the calibrator, was defined by the ΔΔCT method, as previously described (25). Endometrial tissue contributed by a normal patient was used as the calibrator in the following normalization formula: target amount = 2^−ΔΔ*Ct*^, where ΔΔCt = [Ct (target gene sample) – Ct (GAPDH sample)] – [Ct (target gene calibrator) – Ct (GAPDH calibrator)]. The latter was calculated by Light Cycler v4.0 software.

### Protein Extraction and Western Blot Analysis

Protein extracts were prepared using RIPA buffer (Thermo Scientific, Rockford, IL, USA) containing freshly added protease and phosphatase inhibitor cocktail (Thermo Scientific). Concentrations of total cell lysates were measured using BCA protein assay kit (Thermo Scientific). Altogether, 30 μg of total protein were mixed with 5 × sample buffer and heated at 95°C for 5 min. The samples were loaded onto 12% sodium dodecyl sulfate-polyacrylamide gels (SDS-PAGE) and electrotransferred to polyvinylidene fluoride membranes (Millipore Corp, Billerica, MA, USA) using a Trans-Blot apparatus (Bio-Rad, Hercules, CA, USA). The membranes were blocked using 5% non-fat skim milk in Tris-buffered saline solution [10 mmol/L Tris-HCl (pH 7.4) and 0.5 mol/L NaCl), adding Tween-20 (0.1% vol/vol]. Blots were probed using primary antibodies to γ-H2AX (1:2,000; Bethyl Laboratories) and GAPDH (1:5,000; Santa Cruz Biotechnology, Dallas, TX, USA), followed by horseradish peroxidase-conjugated secondary anti-mouse (1:6,000; Thermo Scientific) or anti-rabbit antibody (1:5,000; Thermo Scientific). Protein detection was achieved by enhanced chemiluminescence (Santa Cruz Biotechnology). The experiment was performed in triplicate for analyses, all data shown being representative.

### Immunofluorescence Staining

Immunofluorescence staining of BRCA1 was performed in five participants of each group. Selected cells on glass coverslips were fixed in cold acetone for 10 min, incubated with 1% BSA/PBS for 10 min, and then stained using mouse anti-BRCA1 monoclonal antibody as primary antibody (sc-135732; Santa Cruz Biotechnology) and goat anti-mouse secondary antibody conjugated with Cy3 (Abcam, Cambridge, UK). Fluorescence images from the slides were viewed and captured using a LSM510 microscope (ZEISS, Jena, Germany) and processed using proprietary LSM image software.

### Statistical Analysis

The sample size was calculated to compare mRNA expression of genes related to DNA DSB repair between two groups. A power calculation was performed using PS Power and Sample Size Calculations (Version 3.1.2). Power analysis showed that at least 30 patients would be needed to detect a 30% difference with a significance level of 0.05 and 80% power. Considering 10% drop-out rate, the sample size required per group was 33.

All data (expressed as mean ± SEM) were subjected to Kolmogorov-Smirnov or Shapiro-Wilk test to check for normal distribution and compared using Student's *t*-test or Mann-Whitney *U-*test, as appropriate. Correlations between ovarian *BRCA* mRNA expression and serum AMH levels were assessed using Spearman's correlation coefficient. All computations relied on standard software (SPSS v16.0; SPSS Inc, Chicago, IL, USA), setting statistical significance at *p* < 0.05.

## Results

### Clinical Characteristics

The endometriosis group and controls did not differ significantly in terms of age. However, controls significantly surpassed endometriosis group members in gravidity (2.16 ± 0.31 vs. 1.13 ± 0.23; *p* = 0.009) and parity (1.35 ± 0.17 vs. 0.63 ± 0.15; *p* = 0.003); and serum CA 125 levels were significantly higher in the endometriosis group than in controls (19.03 ± 2.59 vs. 65.27 ± 8.28; *p* < 0.001). BMI was significantly lower in the endometriosis group than in controls (20.47 ± 0.30 vs. 22.39 ± 0.44; *p* < 0.001). All patients in the endometriosis group had advanced-stage endometriosis, showing a mean revised AFS score of 49.22 ± 25.57. Serum AMH levels were significantly lower in the endometriosis group, compared with controls (2.05 ± 0.40 vs. 4.97 ± 1.18; *p* = 0.039) (Table 1).

**Table 1:** Clinical characteristics of study participants with and without endometriosis.

	**EMS (*n* = 38)**	**Control (*n* = 31)**	***p-*value**
Age (years)	35.02 ± 1.22	36.64 ± 1.18	0.353
Gravidity	1.13 ± 0.23	2.16 ± 0.31	0.009
Parity	0.64 ± 0.15	1.35 ± 0.17	0.003
BMI (kg/m^2^)	20.47 ± 0.30	22.39 ± 0.44	< 0.001
CA-125 (U/mL)	65.27 ± 8.28	19.03 ± 2.59	< 0.001
**EMS STAGE**			
III	18 (47.4%)	N/A
IV	20 (52.6%)	
rAFS scores	49.22 ± 25.57	N/A
Serum AMH (ng/mL)	2.05 ± 0.40	4.97 ± 1.18	0.039

*Data expressed as mean ± standard error mean (SEM) BMI, body mass index; EMS, endometriosis; rAFS, revised American Fertility Society; AMH, anti-Müllerian hormone*.

### Immunohistochemical Staining of γ-H2AX

**Figure 1:**
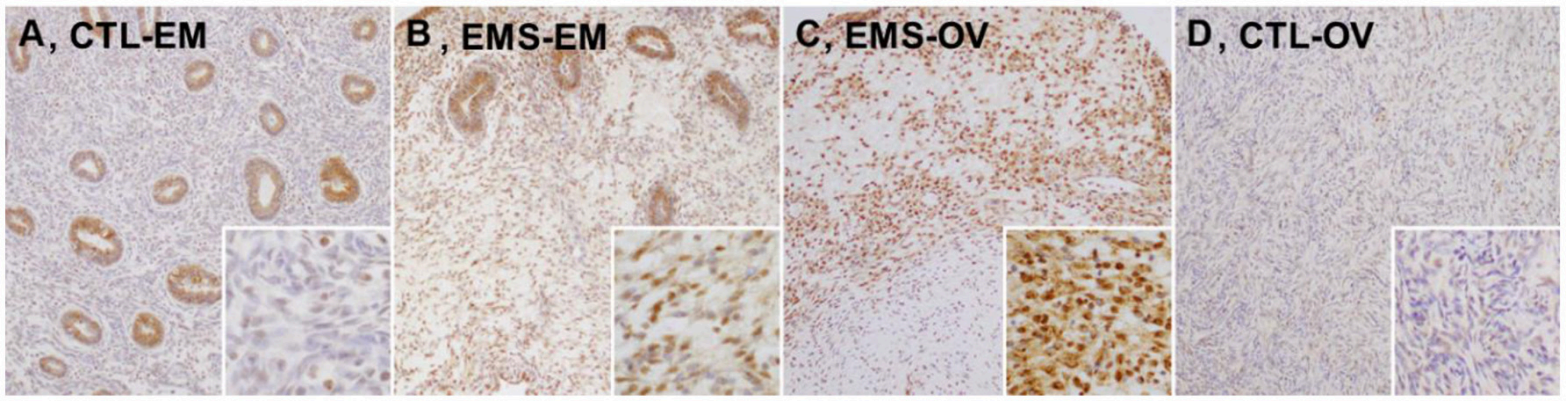
Expression levels of γ-H2AX protein in tissue samples of eutopic and ectopic endometrium of patients with and without endometriosis. **(A)** CTL-EM, eutopic endometrium of control group: γ-H2AX expression absent, or very faint in stromal cells; **(B)** EMS-EM, ectopic endometrium of endometriosis group: patchy γ-H2AX expression of variable staining intensity, most stromal cells showing moderate nuclear γ-H2AX immunoreactivity; **(C)** EMS-OV, ectopic endometrium of endometriosis group: stromal cells demonstrating uniform and strong γ-H2AX immunoreactivity (γ-H2AX expression by glandular epithelium similar in control and in endometriosis groups); and **(D)** CTL-OV, normal ovarian stroma: absence of γ-H2AX expression (original magnification: **A–D**, × 100; inset, × 400).

We evaluated **γ**-H2AX protein expression in eutopic and ectopic endometrial tissues of patients with or without endometriosis in immunostained tissue sections. Representative images **γ**-H2AX immunoreactivities are shown in Figure 1. In eutopic endometrial tissue of the control group, **γ**-H2AX expression was absent or faint in nuclei of stromal cells (Figure 1A); whereas most stromal cells in eutopic endometrium of patients with endometriosis showed moderate nuclear **γ**-H2AX expression (Figure 1B), and some stromal cells showed faint cytoplasmic **γ**-H2AX positivity. In a sample of ovarian endometrioma, **γ**-H2AX expression was clearly increased, compared with eutopic endometrial tissues. Uniform and strong nuclear **γ**-H2AX expression was also observed in ectopic endometrium of patients with endometriosis (Figure 1C). In normal ovarian stroma, no **γ**-H2AX expression was evident (Figure 1D). No between-group difference in glandular expression of **γ**-H2AX was observed.

### Western Blot Analysis of γ-H2AX Expression After H_2_O_2_ Treatment of Cultured Endometrial Stromal Cells and Ishikawa Cell Lines

**Figure 2:**
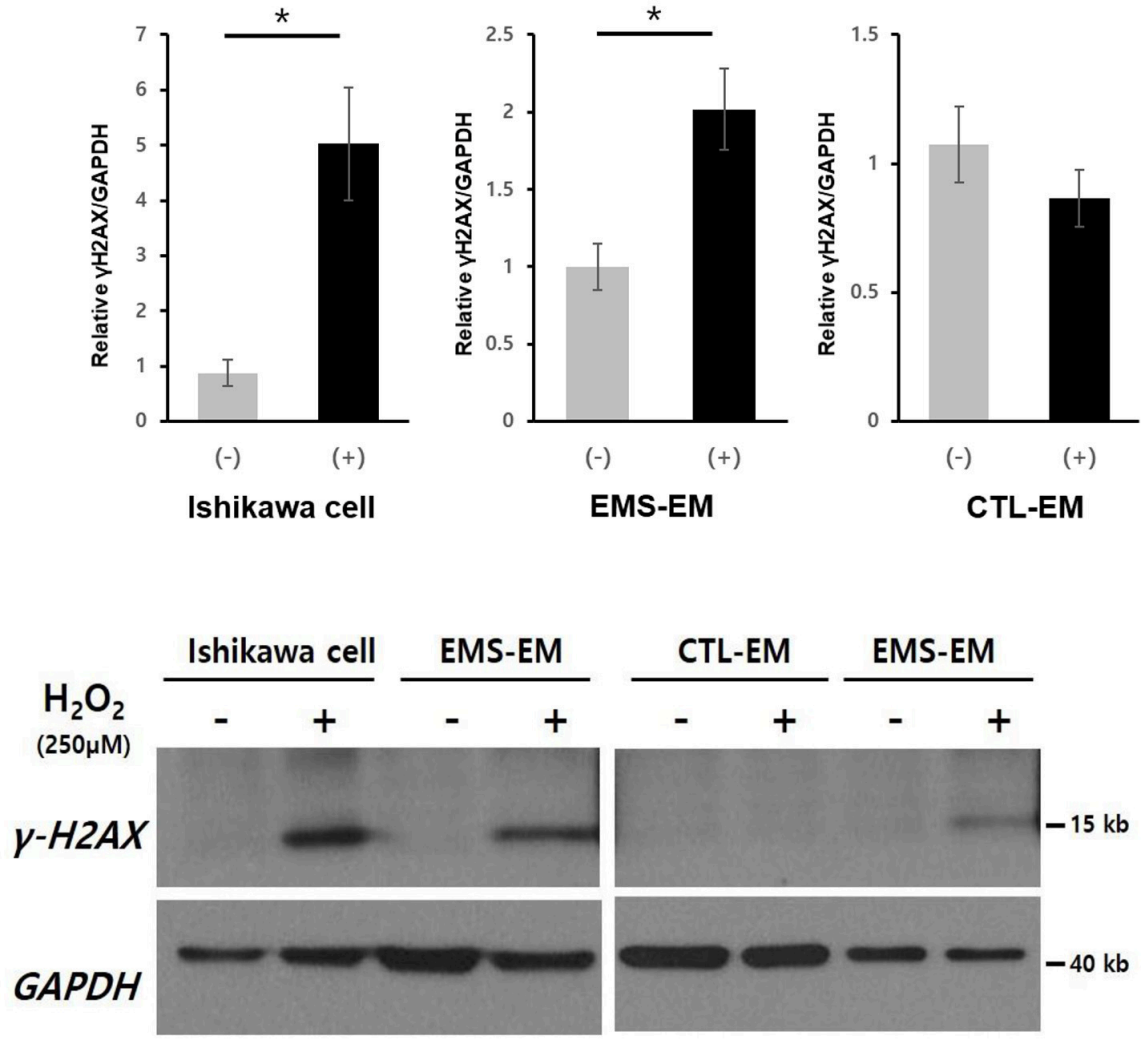
Western blot analysis of γ-H2AX in cultured Ishikawa cell lines and in primary endometrial stromal cells of patients with and without endometriosis after H_2_O_2_ treatment. *p < 0.05. Data are expressed as mean ± SEM values. +, with H_2_O_2_ treatment; –, without H_2_O_2_ treatment; CTL-EM, eutopic endometrium of patients without endometriosis; EMS-EM, eutopic endometrium of patients with endometriosis.

After H_2_O_2_ treatment of cultured Ishikawa cell lines and endometrial stromal cells from patients with endometriosis and controls, **γ**-H2AX expression as a marker of DNA DSB was measured by western blot (Figure 2). Post-treatment **γ**-H2AX expression levels in Ishikawa cells and endometrial stromal cells of patients with endometriosis patients were significantly elevated, compared with pretreatment levels [relative **γ**-H2AX expression/GAPDH: 0.88 ± 0.25 vs. 5.03 ± 1.02 (*p* = 0.049) and 1.00 ± 0.15 vs. 2.02 ± 0.26 (*p* = 0.016), respectively]. However, no **γ**-H2AX expression was evident in endometrial stromal cells of controls, despite observed H_2_O_2_ treatment (relative **γ**-H2AX expression/GAPDH: 1.07 ± 0.15 vs. 0.86 ± 0.11; *p* = 0.32).

### Expression Levels of *BRCA1, BRCA2, Rad51*, and *ATM* mRNA in Endometriosis Group and Controls

**Figure 3:**
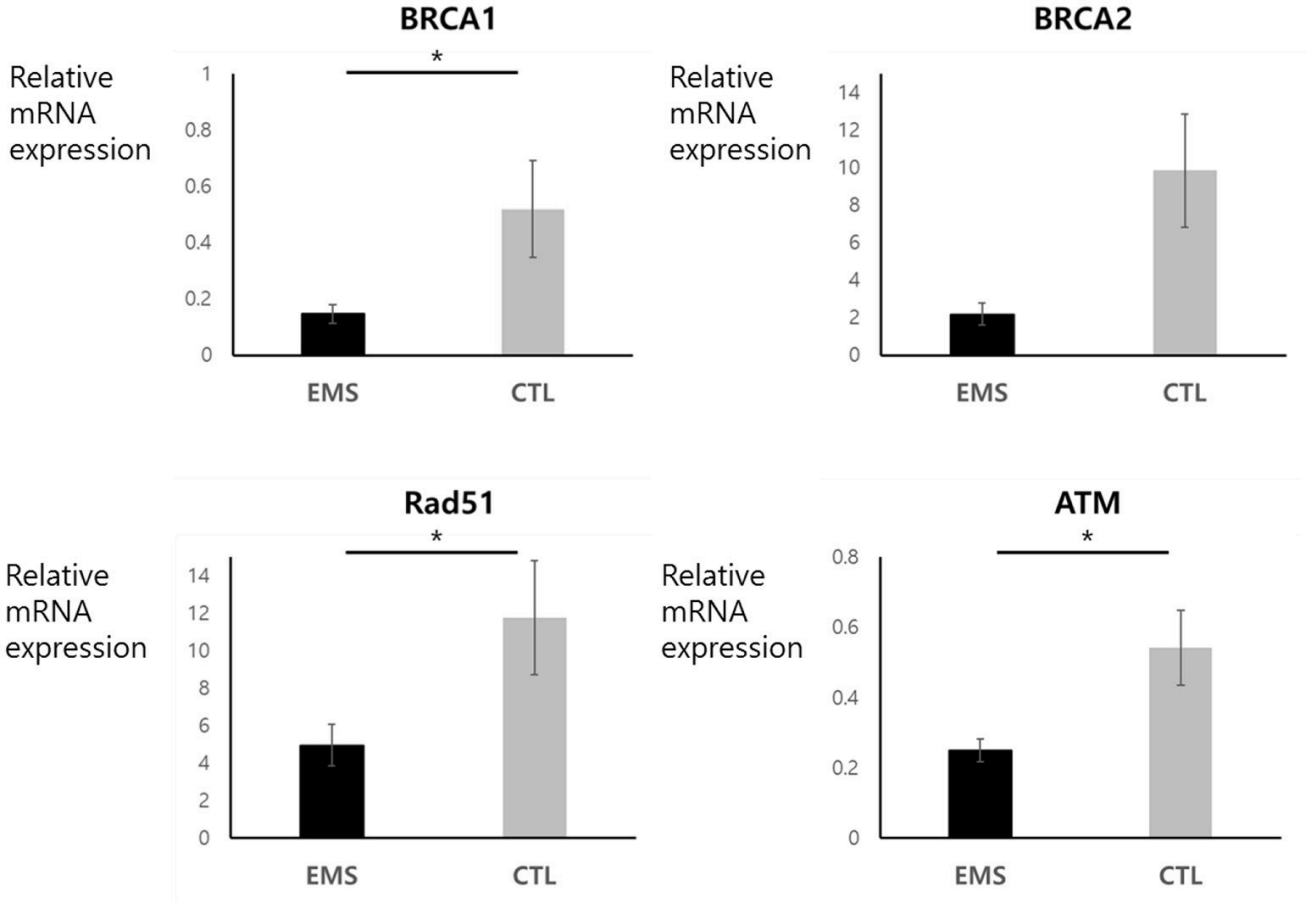
Endometrial mRNA expression of *BRCA1, BRCA2, Rad51*, and *ATM* in endometriosis group and in controls. *p < 0.05. Data are expressed as mean ± SEM.

**Figure 4:**
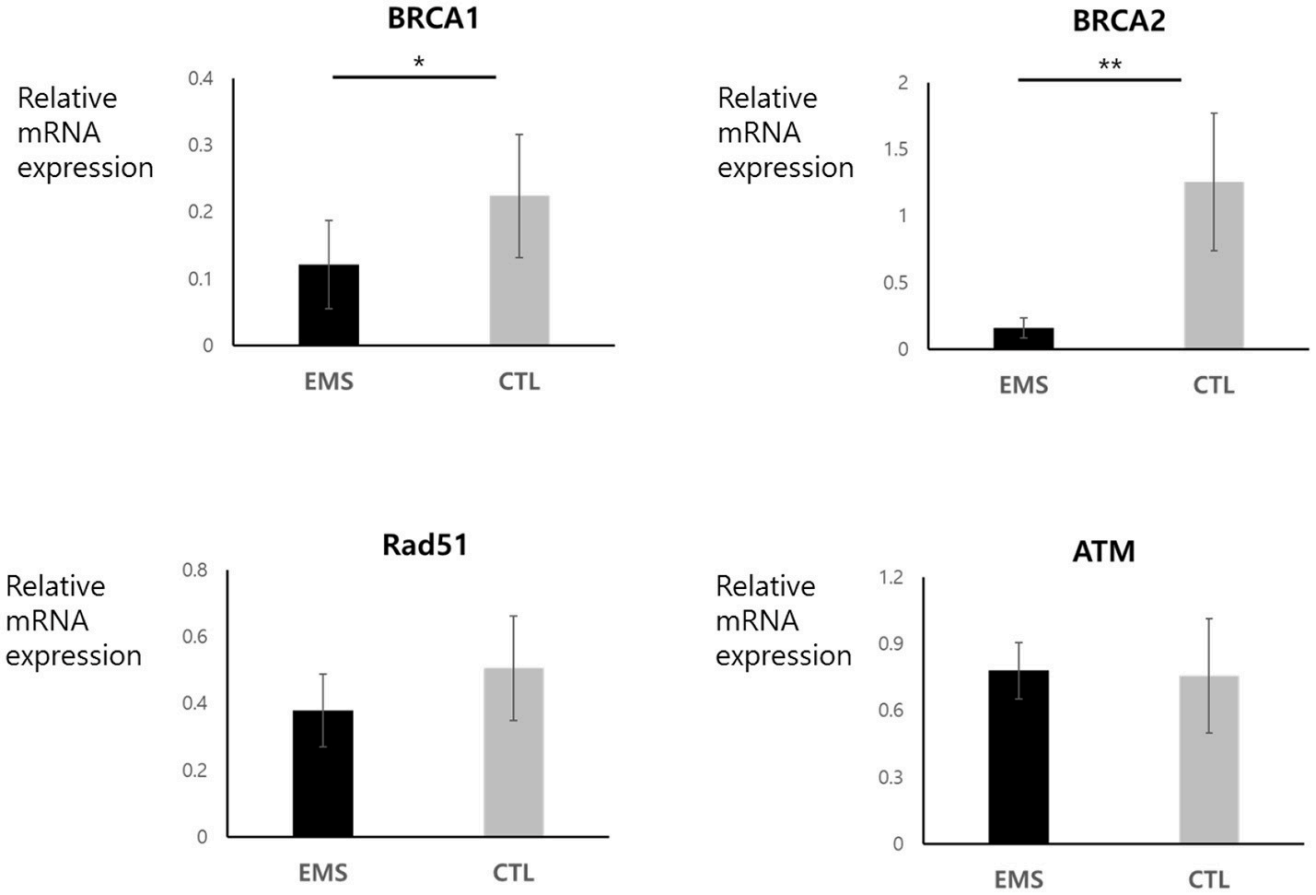
Ovarian mRNA expression of *BRCA1, BRCA2, Rad51*, and *ATM* in endometriosis group and in controls. *p < 0.05, **p < 0.01. Data are expressed as mean ± SEM.

Endometrial and ovarian expression levels of *BRCA1, BRCA2, Rad51*, and *ATM* mRNA in the endometriosis group and in controls are shown in Figures 3, 4. Although endometrial *BRCA2* mRNA expression was comparable in both groups (2.19 ± 0.59 vs. 9.86 ± 3.02; *p* = 0.051), levels of *BRCA1, Rad51*, and *ATM* were significantly lower in endometriosis group, compared with controls [0.25 ± 0.03 vs. 0.54 ± 0.11 (*p* = 0.014); 4.95 ± 1.11 vs. 11.77 ± 3.05 (*p* = 0.024); and 0.25 ± 0.03 vs. 0.545 ± 0.11 (*p* = 0.016), respectively]. Ovarian expression of *BRCA1* and *BRCA2* was significantly lower in the endometriosis group, compared with controls [0.12 ± 0.06 vs. 0.22 ± 0.09 (*p* = 0.045) and 0.16 ± 0.08 vs. 1.26 ± 0.52 (*p* = 0.001), respectively]; but *Rad51* and *ATM* expression levels were comparable in the two groups [0.38 ± 0.11 vs. 0.51 ± 0.16 (*p* = 0.621) and 0.79 ± 0.13 vs. 0.76 ± 0.26 (*p* = 0.526), respectively].

**Figure 5:**
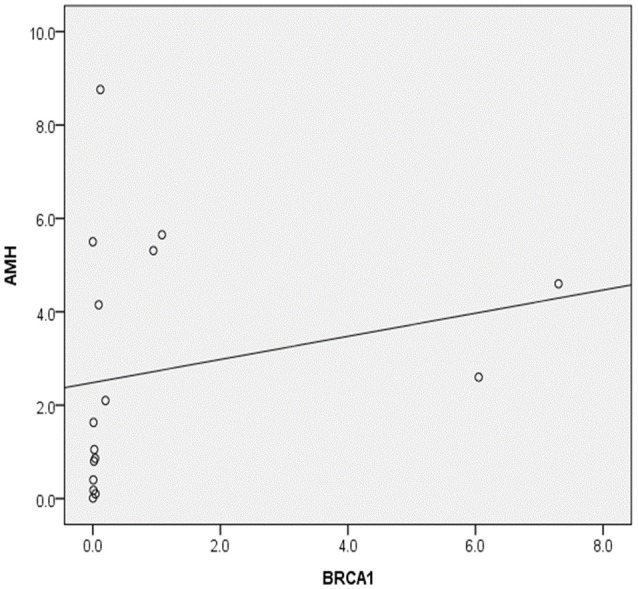
Correlation between serum anti-Müllerian hormone levels (AMH) and ovarian *BRCA1* expression in patients with endometriosis (Spearman's rho, 0.541; *p* = 0.030).

Serum AMH concentration and ovarian *BRCA1* mRNA expression correlated significantly in the endometriosis group (correlation coefficient, 0.541; *p* = 0.03) (Figure 5).

### Immunofluorescence Staining of *BRCA1*

**Figure 6:**
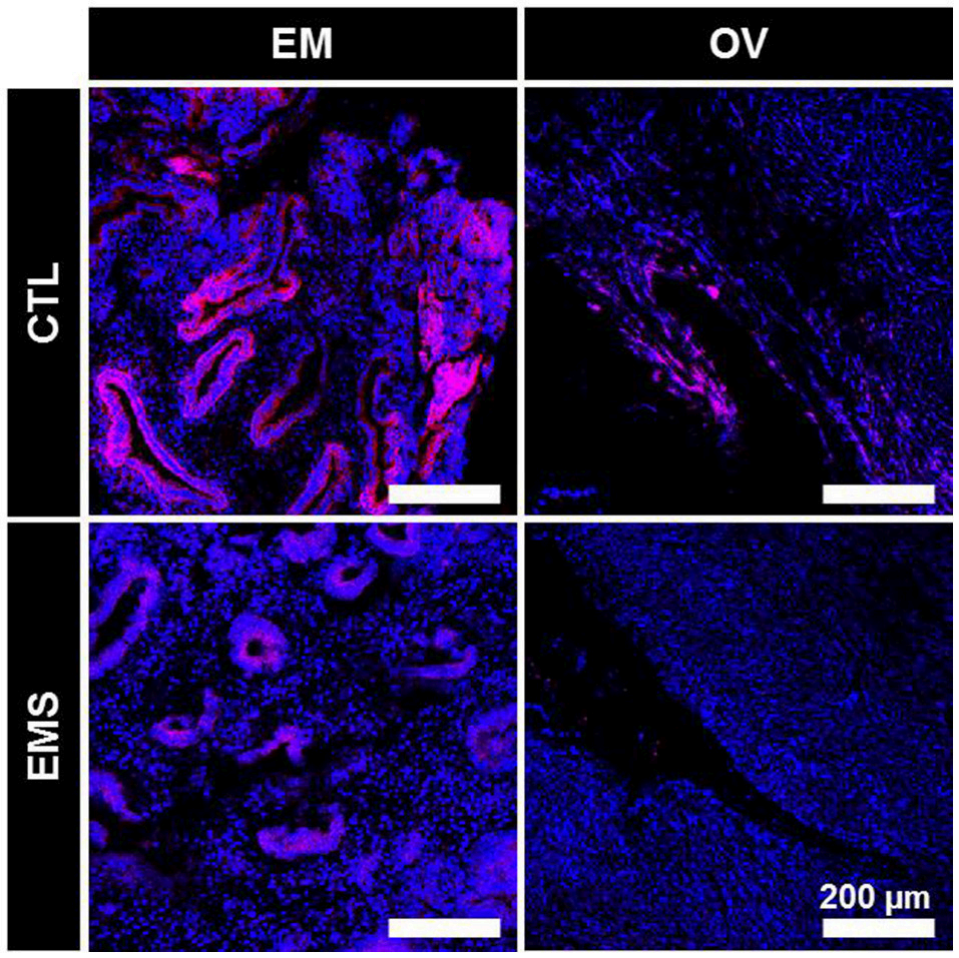
Immunofluorescence staining of BRCA1 in eutopic endometrium and ovarian tissue of patients with and without endometriosis. CTL-EM, eutopic endometrium of patients without endometriosis; EMS-EM, eutopic endometrium of patients with endometriosis; CTL-OV, Non-endometriotic ovarian tissue; EMS-OV, ovarian endometriotic cyst.

Decreased expression of BRCA1 protein in endometrial and ovarian tissues of the endometriosis group was confirmed by immunofluorescence staining (Figure 6).

## Discussion

In the present study, we have demonstrated that mRNA expression levels for key genes related to DSB repair are reduced in women with endometriosis. Indeed, expression levels of *BRCA1, Rad51*, and *ATM* in endometrial tissue proved to be lower in the endometriosis group than in control subjects, as were expression levels of *BRCA1* and *BRCA2* in ovarian tissue. Ovarian BRCA1 expression in particular correlated with serum AMH levels as a marker of ovarian reserves. To our knowledge, this is the first study to evaluate the role of impaired DNA DSB repair in the pathogenic declines of ovarian reserves experienced by women with endometriosis.

Above findings are aligned with previous studies of diminished ovarian reserves in women with *BRCA* mutations. Women with germline mutations of the *BRCA1* gene showed low responses to controlled ovarian stimulation for fertility preservation by oocyte or embryo cryopreservation (26) and had lower age- and BMI- adjusted serum AMH levels, indicating that *BRCA1* mutations may be associated with decreased ovarian reserves. Furthermore, women with *BRCA1* mutations may also be associated with earlier menopause (27, 28). In a recent comparative study of ovarian tissue obtained from unaffected *BRCA* mutation carriers and age-matched cadaveric organ donors, *BRCA* mutations were associated with reduced ovarian reserves as well as accelerated loss of primordial follicle and oocyte DNA damage (29). These observations support that DNA DSB repair plays an important role in maintaining ovarian reserve and suggest that such reserves are prematurely depleted in women with *BRCA1* mutations.

Apart from *BRCA1/2* germline mutations, other BRCA defects (including methylation of the *BRCA1* promoter, low-level expression, and copy-number deletions) in some sporadic cancers share phenotypic traits of tumors that carry BRCA1*/2* mutations (30). *BRCA1/2* mRNA expression levels have been previously investigated in women with breast and ovarian cancers (31, 32), suggesting that they correspond with survival rates or chemotherapeutic sensitivity. Although the present study was aimed at mRNA expression levels of genes related to DNA DSB repair, faltering mRNA expression of these genes may affect ovarian reserves in a manner similar to women with *BRCA* germline mutations. It is believed that *BRCA1*-related DNA DSB repair efficiency may be an important determinant of oocyte aging in women (17). Researchers have found that *BRCA1* gene expression showed a significant age-related decline in oocytes and that oocyte-specific knockdown of *BRCA1* expression increases DSBs.

Endometriosis is a disease related to elevated oxidative stress in the follicular environment, paracrine environment, and systemically (33). Oxidative stress causes DNA damage (34). Histone H2AX, one of several variants of the nucleosome core histone H2A, becomes phosphorylated on Ser139 in response to DSBs (γ-H2AX). Within seconds of DSB occurrence, γ-H2AX foci appear at sites of DNA damage, which are detectable by confocal microscopy, or immunohistochemistry in quantifying DNA damage. Foci of γ-H2AX represent DSBs in a 1:1 ratio, enabling sensitive quantitation of DSBs (35).

In the present study, we found that occurrences of DNA DSBs, as represented by γ-H2AX protein expression, increased in endometrial, and ovarian endometrioma samples of patients with endometriosis. However, two earlier immunohistochemical studies have reported results contradictory to ours. In women with endometriosis, persistence of proliferative markers in eutopic endometrial cells seemed to be associated with virtually complete loss of γ-H2AX (36); and endometrial cells from ectopic sites dysplayed immune-staining for proliferative markers, with concomitant loss of the γ-H2AX staining in ectopic endometriotic lesions of both human and baboon endometriosis model (37). These differences may be due to the differing characteristics of subjects selected for study. Although all of our patients had advanced-stage endometriosis with endometrioma(s), all stages of peritoneal endometriosis (I-IV) were included. Moreover, immunnostains performed in the latter study were confined to peritoneal endometriotic lesions. Immunodetection of γ-H2AX is used to quantify DNA damage in cells and tissues, and has diagnostic and prognostic value in cancer. High phosphorylation levels are indicative of defective DNA repair and genomic instability in premailignant lesions and in tumors; and they are associated with higher-grade malignancy and poor prognoses in various cancers, including breast, colorectal, lung, and ovarian cancers, and melanoma (38) as well as endometrial cancer (39).

In our experiments with *in vitro* cell cultures, we also demonstrated that endometrial and ovarian tissues of patients with endometriosis are more vulnerable to genotoxic stimuli, showing an increased propensity for DNA damage compared with controls. Following genotoxic exposure with H_2_O_2_, γ-H2AX expression by endometrial stromal cells, and Ishkawa cell line increased in tandem, whereas endometrial stromal cells of control subjects were devoid of γ-H2AX expression, even after H_2_O_2_ treatment. These findings may certainly stem from impaired DNA DSB repair.

Although we did not directly demonstrate attenuated DNA DSB repair or DSB repair mechanisms in ovarian follicles or oocytes, we did establish that *BRCA1* expression in ovarian endometriotic tissue and serum AMH level (a marker of ovarian reserve) correlated significantly. Diminished expression of *BRCA* in ovarian endometrotic tissue is then indicative of impaired DNA DSB repair and ultimately is attributable to follicular damage. Given that endometriosis is associated with oxidative stress locally and systemically and incite chronic inflammation within the peritoneal cavity, heightened DNA damages, and impairment of DNA repair in ectopic endometrial tissue may mirror the status of ovarian follicles, both oocytes and surrounding follicular cells. Finally, pathologic findings akin to chemotherapy-induced ovarian damage (i.e., focal fibrosis, vascular deficiency, and concomitant lowering of follicular density) in women with endometriosis may attest to microvascular and stromal damage. As documented in the mouse ovary, doxorubicin insult initially induces DNA damage in stroma, theca, and granulosa cells, followed by oocytic DNA damage (40). Thus, DNA damage in adjacent somatic cells and stroma also plays a role in decreased ovarian reserves.

Although gene expression patterns of endometrium might be different according menstrual phase, we compared gene expression patterns irrespective of menstrual phase. However, their distribution in the present study was comparable between the two groups. When we also compared mRNA expression of BRCA1, BRCA2, Rad 51, and ATM between proliferative phase and secretory phase endometrium using quantitative RT-PCR, there were no significant differences in mRNA expressions of BRCA1, BRCA2, Rad 51, and ATM between proliferative and secretory phase endometrium (data were not shown).

Eutopic endometrium of women with endometriosis shows fundamental differences compared with that of healthy control (23). The eutopic and ectopic endometrium of women with endometriosis shares alterations that are not found in the eutopic endometrium of women without endometriosis, corroborating the idea that this altered endometrium in the peritoneal cavity has the initial potential to develop endometriosis (41). In the present study, we observed increased DNA damages and impaired DNA DSB repair mechanism in eutopic endometrium as well as ectopic endometrium. However, there were no significant correlations between genes related to DNA DSB repair in eutopic endometrium and AMH. Although it could not be clearly explained, it may due to small sample size. On the other hand, it may be explained as a direct consequence of the different endocrine microenvironments such as the peritoneal fluid and the intraovarian microenvironment of ectopic endometrium and the intrauterine environment in eutopic endometrium. Nonetheless, considering that autocrine and paracrine effects following DNA damage, findings of ectopic endometrium may be more important than those of eutopic endometrium with respect to ovarian reserve.

In the clinical characteristics, BMI was significantly lower in endometriosis group compared with controls. Oxidative stress in obesity poses a significant threat to DNA stability and integrity as indicated by the growing number of investigations reporting a positive correlation between markers of oxidative DNA damage and increased adiposity (42). Obesity induces DNA damage in hematopoietic stem cell transplant recipients that have been treated by cyclophosphamide (43). Since there were significant increases in DNA damages and impairment of genes related DNA DSB repair in spite of protective effect of low BMI in endometriosis group, the difference of BMI could not affect our results.

In conclusion, we have determined that expression levels of various genes implicated in DSB repair are diminished in patients with endometriosis. In particular, *BRCA1* expression is decreased in both ovarian and endometrial tissues of such patients and appears to correlate with AMH, a marker of ovarian reserves. These findings indicate that impaired DSB repair is a likely contributor to diminished ovarian reserves in women with endometriosis. However, further studies will be necessary to confirm the association between impairment of DNA DSB repair and decreased ovarian reserve in this setting.

## Author Contributions

YC, SC, and H-SK contributed to study conception and design, acquisition, analysis, and interpretation of data and drafting of the manuscript. JiP, JL, J-KY, BY, JoP, SS, and H-JS aided in acquisition and analysis of data for the work. BL contributed to conception and design. All authors participated substantially in these research efforts and then critically appraised, revised, and approved the manuscript.

### Conflict of Interest Statement

The authors declare that the research was conducted in the absence of any commercial or financial relationships that could be construed as a potential conflict of interest.
